# Evaluation of Early Neuro-Imaging Requests for Dementia Diagnosis in Wolverhampton Memory Assessment Service (MAS)

**DOI:** 10.1192/bjo.2022.401

**Published:** 2022-06-20

**Authors:** Aparna Prasanna, Kuljit Mandair, Clare Ling

**Affiliations:** Black Country Healthcare NHS Foundation Trust, Wolverhampton, United Kingdom

## Abstract

**Aims:**

The Wolverhampton Memory Assessment Service (MAS) is nurse led and accepts referrals from primary and secondary care settings. There has been a rapid rise in the number of referrals as well as an increase in demand to provide a timely diagnosis. This poses a challenge to meet the national aspiration of referral to diagnosis in 6 weeks. The aim is to improve access to neuroimaging in order to avoid delays to diagnosis and management.

**Methods:**

In January 2022, a retrospective sample of three groups of newly referred patients to MAS between 1st June-31st October 2021 was selected, each group consisting of 15 patients.

A dedicated tool was used to collect data. MAS follows NICE standards for neuroimaging in dementia guidance.

In Group 1 scans were not requested at referral but were requested after initial nursing assessment, in Group 2 scans were available at initial referral and in Group 3 scans were requested by the MAS Consultant Psychiatrist upon receipt of referral.

**Results:**

In group 1; 47% of patients have still not had a scan (with a waiting time of approximately 6 months) and 73% have not been given a diagnosis. Three patients were given a diagnosis due to exceptional circumstances and therefore the results of these patients can be disregarded.

In group 2, all (100%) patients had a scan either prior to the referral (73%) or requested by GPs at the time of referral (27%). 80% of patients have been given a diagnosis. The average days from referral to diagnosis was 82 days. Patients not given a diagnosis yet was due to cancellation/awaiting appointments.

In group 3, all (100%) patients have had a scan and 67% of patients have been given a diagnosis. The average days from referral to diagnosis was 102 days. Patients not given a diagnosis yet was due to cancellation/awaiting appointments.

**Conclusion:**

Implementing a pathway whereby clinicians can either have access to prior neuroimaging or refer appropriate patients for scans at the point of referral, significantly reduces waiting times to diagnosis and management within a timely manner.

This reduces carer burden and provides increased support from appropriate services as well as reducing the chances of patients ending up on crisis pathways.

There is a need to implement an integrated care pathway that is responsive and accessible to all patients.

